# All-inclusive nitrifiers in Antarctic soils

**DOI:** 10.1038/s41467-024-47441-y

**Published:** 2024-04-12

**Authors:** Maximiliano Ortiz

**Affiliations:** Clemson University Genomics & Bioinformatics Facility, Clemson University, Clemson, USA

**Keywords:** Microbial ecology, Metagenomics, Bacterial genetics, Element cycles

## Abstract

Multidisciplinary culture-dependent and -independent techniques elucidate the unique microbial nitrogen cycle in nutrient-poor coastal Antarctica soils and reveal the contribution of novel key microbes to their nitrogen budget.

New research challenges require the combination of multidisciplinary approaches to describe intricate biological processes, and the most recent microbiome discoveries in the ice-free areas of the Antarctic continent clearly confirms this. Antarctica is considered one of the harshest environments on Earth with severe freeze-thaw cycles, katabatic winds, high UV, thermal and a profound limitation for water, nutrients, and particularly organic carbon and nitrogen^1^. The ice-free regions that cover only 0.44% of the continental land are not an exception^2^. Life in these regions is dominated by microorganisms that carry out most of the nutrient cycling and ecosystem servicing processes. It might be surprising that these extreme environments with poor nutrient content harbor a high diversity of microorganisms comparable to that of temperate zones in the middle latitudes of the globe^3,4^. Building on this premise, the main question that arises is: What are the metabolic strategies used by microbial communities to thrive in this oligotrophic extreme environment? To start answering this question, researchers need to face a common conundrum in Antarctic microbial ecology: if the extreme conditions allow some microbial growth, this phenomenon occurs at a slow rate in specific time windows. Hence, a combination of cutting edge methods is necessary to capture the microbial composition and measure their metabolic activity/ies during the active phase of the prokaryotic cells, or even at dormant states where the cell requires a minimum energy input to survive (Fig. 1).

**Fig. 1:**
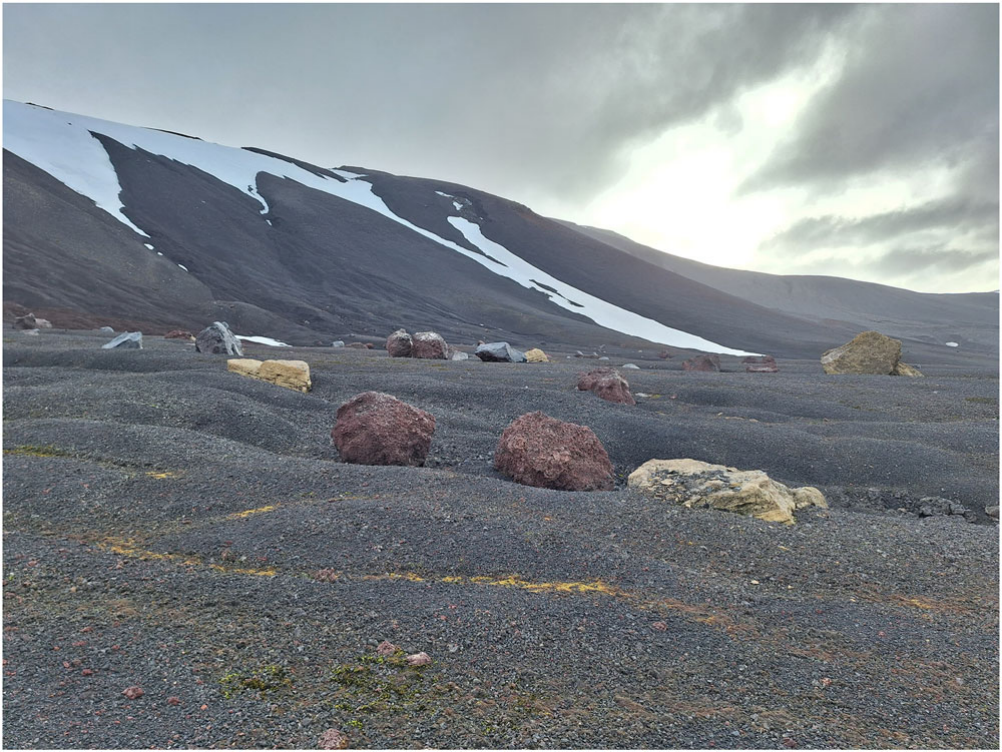
Antarctic oligotrophic soils. Han et al.^6^ report the analysis of microbial communities in surface sediments and water, and surface soils near the shore of different lakes in the Larsemann Hills area, the second-largest ice-free land in East Antarctica. Their results describe a unique microbial N-cycling dynamic and identify previously undetected bacteria as key players in the N-budget in this extreme environment. Credit: Prof. Jean-Baptiste Ramond. Pontificia Universidad Católica de Chile. (INACH Project RT_05_22 / ECA 60).

## Bridging the gap with multidisciplinary approaches

The constant improvement of multidisciplinary methodologies over the years is allowing scientists to achieve this analytical level to profile the functional capabilities and the taxonomic composition of extreme microbiomes. These methods include a variety of culture independent and culture dependent techniques that are worth mentioning briefly. By definition, culture dependent techniques are based on the isolation and cultivation of microorganisms under controlled conditions while independent methods rely mainly on the direct analysis of nucleic acids (DNA and RNA), other biomolecules or metabolic products. Considering that a restricted percentage of microorganisms can be grown in the laboratory, Antarctic culture-based studies have isolated a limited number of bacteria from a few bacterial groups that do not represent the complexity and diversity of the actual microbiome.

Therefore, the implementation of culture-independent approaches and the use of simplified ecosystems under controlled conditions, known as microcosms, are making a significant impact on studying the dynamics of microbial communities in polar environments. In this regard, advanced high-throughput DNA sequencing is enabling scientists to recover high-quality microbial draft genomes and their gene content from complex samples. However, the mere presence of genomic material does not conclusively indicate whether the microbes and their genes are actively participating in ecosystem processes. This is where functional assay approaches become indispensable. These approaches play a crucial role in detecting and measuring the metabolic rates and activities within the communities, thereby complementing DNA sequence-based methodologies.

By applying these state-of-the-art methods in the last decade, unprecedented microbial metabolic strategies of energy and nutrient acquisition have started to be disentangled. Now we are aware that Antarctic soil bacteria are metabolically versatile aerobes able to generate energy, biomass and even water using atmospheric gases such as hydrogen and carbon monoxide, independently of the classic photosynthetic microorganisms (Cyanobacteria and microalgae)^3,4^. Nevertheless, bacteria using this widespread “living on air” strategy coexist with microorganisms that adopt a wide range of other nutritional and ecological lifestyles such as harvesting solar energy, oxidizing edaphic inorganic substrates, or adopting symbiotic associations^4^. However, there are some critical gaps in our knowledge regarding Nitrogen cycling and nitrogen processing in Antarctic soils. While the distribution of N-fixing, N-processing organisms and N-cycling genes is starting to be quite comprehensive, the limited data on N-processing rates represents a bottleneck in modeling the nitrogen turnover contribution to ecosystem services^5^.

## New research uncovers a key nitrogen driver

Thanks to the insightful work performed by Han and colleagues, our conception about N-cycling in Antarctica is about to change^6^. The authors used a combination of advanced genomic analyses, quantification of N-functional genes, physicochemical analysis, isotopic assays and microcosms incubation to unveil a unique microbial N-cycling dynamic and identify previously undetected bacteria as key players in the N-budget in the extreme, poor-nutrient coastal Antarctica. They focused their work on surface sediments and water, and surface soils near the shore of six lakes in the Larsemann Hills area, the second-largest ice-free land in East Antarctica. First, and by implementing isotopic signatures analyses, the authors determined that the primary production of nitrate can be largely attributed to the microbial nitrification activity instead of atmospheric precipitation as previously thought. Additionally, they extensively quantified the N-cycling functional genes to reveal a comparable amount of N-fixation and nitrification genes present in the microbial communities, with higher relative abundances for denitrification genes. Surprisingly, this quantitative evaluation of nitrification-related functional genes established that ammonia-oxidizing archaea (AOA) and unexpected comammox bacteria were the predominant nitrifiers. Here, the term “comammox” refers to COMplete AMMonia-OXidizing, associated to the recently described *Nitrospira* bacteria genera^7,8^ and unreported previously in Antarctic ecosystem, capable of performing the entire nitrification process on its own. No anaerobic ammonia-oxidizing functional markers were detected, suggesting the uniqueness of N-cycling dynamic in this region.

Building on these findings, the authors obtained DNA-sequence datasets and applied advanced high-throughput DNA sequencing and bioinformatics techniques to recover the draft genome sequences of a couple thousand key members of the microbial communities. These genomes span 29 major groups (phyla) of prokaryotes and represent the most comprehensive inventory of Antarctica coastal soil and sediment genomes to date, surpassing the previous inventory reported in the McMurdo Dry Valleys^4^. These invaluable genomics resources will facilitate future research regarding the complex relationships in Antarctic edaphic microbial communities, their biogeochemical cycling capacities, and ultimately on how life has evolved and adapted in polar regions and their response to climate change. Based on the functional profiling of recovered genomes, dominant members of the community seem to rely on aerobic respiration and have the potential to use different energy conservation and carbon acquisition pathways. Also, as described above and reported previously^3,4^, the most abundant and widespread microbes encode trace gas oxidation genes. These observations confirm that different metabolic strategies facilitate the generation of biomass separately or complementary to photosynthetic metabolism. In terms of the metabolic capacity within the genomes to process nitrogen, N-fixation appears to be carried out by five bacterial clades, while the potential for denitrification is present across all recovered genomes, which further highlights the importance of cold-adapted denitrifiers in Antarctic ecosystem N-cycle.

In line with the quantification of nitrification-related genes, only AOA and *Nitrospira* genomes were identified among the nitrifying groups. To complement these results and by using exclusively nitrification genes as markers, the authors screened the abundance and community structure of all identified nitrifiers. Surprisingly, they confirm the unexpected dominance of one specific *Nitrospira* group, the clade B comammox *Nitrospira*. This indicates a pivotal role of this specific *Nitrospira* as a novel nitrification driver in Antarctic soils. Among the recovered and highly-abundant *Nitrospira* genomes, two were associated to the clade B comammox. While one was related to a genome recovered from Arctic permafrost, the other belonged to a new and unique group within clade B. This further shows that the use of high throughput sequencing technologies and bioinformatics on the various biotopes and niches of the white continent are necessary to fully apprehend its microbial diversity and functioning.

Nevertheless, DNA-sequence analyses are not sufficient to assess the activity of microorganisms within a given environment. Therefore, to evaluate the activity of comammox bacteria in the collected lake sediments and soil samples, Han and colleagues tracked the fate of carbon isotopic labeled substrates in controlled microcosm experiments at 4 °C and 10 °C and confirmed the active role of comammox Nitrospira as a key drivers of nitrification in coastal East Antarctica open soils. Besides this fascinating metabolic strategy, clade B comammox Nitrospira harbor the genetic potential to rely on different adaptive tactics as described above. Altogether, these results strongly suggest that clade B species might have superior affinity for their substrate, and are potentially best adapted to thrive in this extreme, poor-nutrient environment.

## Looking ahead

Despite the conclusive results presented in this work, new inquiries as well as new research opportunities emerge. For instance, N-fixing Cyanobacteria are scarce or confined to specific lithic niches in more nutrient-deprived Antarctic edaphic environments such as in the McMurdo Dry Valleys. In this context, several questions arise: How can nitrification work in environments where Cyanobacteria are virtually absent and no ammonium is bioavailable? Is there a complete microbial-lead N-cycle in such environments where nitrification is virtually absent? Are other bacteria responsible for these atmospheric N-fixation and nitrification processes? Is *Nitrospira* the primary nitrification driver across other biogeographical Antarctic zones? What is/will be the N-dynamic in warming conditions in the current climate change scenario? There is no doubt that advanced multidisciplinary approaches are needed to address these complex questions and continue advancing the fields of environmental microbiology and microbial ecology.
